# NLRP3 Mediates NF-κB Activation and Cytokine Induction in Microbially Induced and Sterile Inflammation

**DOI:** 10.1371/journal.pone.0119179

**Published:** 2015-03-11

**Authors:** Takeshi Kinoshita, Ryu Imamura, Hiroko Kushiyama, Takashi Suda

**Affiliations:** Division of Immunology and Molecular Biology, Cancer Research Institute, Kanazawa University, Kakumamachi, Kanazawa, Ishikawa, Japan; University of California Merced, UNITED STATES

## Abstract

Nucleotide-binding domain and leucine-rich repeat-containing family, pyrin domain containing 3 (NLRP3) has recently emerged as a central regulator of innate immunity and inflammation in response to both sterile inflammatory and microbial invasion signals. Although its ability to drive proteolytic procaspase-1 processing has drawn more attention, NLPR3 can also activate NF-κB. To clarify the physiological relevance of this latter function, we examined the effect of NLRP3 on NF-κB activation and cytokine induction in RNA-interference-based NLRP3-knockdown cell lines generated from the human monocytic cell line THP-1. Knocking down NLRP3 reduced NF-κB activation and cytokine induction in the early stages of *Staphylococcus aureus* infection. Expression of cytokine genes induced by *Staphylococcus aureus* was not inhibited by a caspase-1 inhibitor, and did not occur through an autocrine mechanism in response to newly synthesized cytokines. We also demonstrated that NLRP3 could activate NF-κB and induce cytokines in response to sterile signals, monosodium urate crystals and aluminum adjuvant. Thus, NLRP3 mediates NF-κB activation in both sterile and microbially induced inflammation. Our findings show that not only does NLRP3 activate caspase-1 post-translationally, but it also induces multiple cytokine genes in the innate immune system.

## Introduction

Nucleotide-binding domain and leucine-rich repeat-containing family, pyrin domain containing 3 (NLRP3, also called cryopyrin, NALP3, and PYPAF1, induces inflammatory responses and cell death in response to various danger signals. These signals include pathogen-associated molecular patterns (PAMPs), such as bacterial and viral RNA and muramyl dipeptide [1–7]; damage-associated molecular patterns (DAMPs), such as ATP, uric acid crystals, cholesterol crystals, and amyloid-β [8–15]; and environmental inflammatory substrates such as asbestos and silica [16, 17]. Thus, NLRP3 is implicated not only in infectious diseases, but also in sterile inflammation in conditions such as gout, atherosclerosis, Alzheimer’s disease, asbestosis, and silicosis. Gain-of-function NLRP3 mutations cause a subset of autoinflammatory diseases collectively called cryopyrin-associated periodic syndrome (CAPS) [18, 19]. When activated, NLRP3 joins with caspase-1 and the adaptor molecule apoptosis-associated speck-like protein containing a caspase recruitment domain (ASC) to form a multi-protein complex called the inflammasome, which controls caspase-1 activation. Activated caspase-1, in turn, cleaves pro-IL-1β and pro-IL-18 into their biologically active secretory forms [20–22].

For macrophages to produce mature IL-1β, two signals are required. The first (signal 1) activates NF-κB and induces pro-IL-1β synthesis, and the second (signal 2) activates caspase-1 and induces the proteolytic maturation of pro-IL-1β. Since NF-κB activation in response to PAMP stimulation and bacterial infection is normal in macrophages from *NLRP3* ^−/−^ or *ASC* ^−/−^ mice [12, 13], NLRP3 has been considered dispensable for signal 1 in mice. While the TLR pathway is generally believed to mediate signal 1 in the case of pathogen-stimulated macrophages, it is not clear how this signal is mediated in sterile inflammation.

Genetic reconstitution experiments using HEK293 cells have shown that human NLRP3 can activate NF-κB [23, 24] in cooperation with ASC. ASC-deficient human monocytic THP-1 cells show defective NF-κB activation and cytokine production in response to *Porphyromonas gingivalis* infection [25]. However, the role of human NLRP3 in activating NF-κB, especially under sterile inflammatory conditions, has not been examined.

To address human NLRP3's role in the NF-κB signaling pathway, we generated THP-1-derived NLRP3-knockdown cells and demonstrated that NLRP3 mediates NF-κB activation and cytokine gene induction in a caspase-1 independent manner. More importantly, NLRP3 could activate NF-κB and induce cytokines following stimulation by monosodium urate (MSU) crystals and aluminum adjuvant, sterile activators for NLRP3. These results suggest that NLRP3 is important for NF-κB activation, especially in sterile inflammatory diseases.

## Methods

### Reagents

Doxycycline (Dox) was purchased from AppliChem (Darmstadt, Germany), recombinant human IL-1Ra from Genzyme (Cambridge, MA, USA), recombinant human IL-1β from PeproTech (London, UK), Ac-YVAD-CMK from Bachem (Torrance, CA, USA), bafilomycin A1 from Fermenteck (Jerusalem, Israel), and CA-074Me from Merck (Darmstadt, Germany). Diphenyleneiodonium (DPI) was purchased from Enzo (Plymouth Meeting, PA, USA). LPS from Escherichia coli K235 was purchased from Sigma-Aldrich (St. Lois, MO, USA). ATP was purchased from YAMASA CORPORATION (Chiba, Japan). Cycloheximide (CHX) and MSU crystals were purchased from Wako Pure Chemical Industries (Osaka, Japan). Aluminum adjuvant (Imject Alum) was purchased from Pierce (Rockford, IL, USA). Ficoll-Paque PLUS was purchased from GE Healthcare Japan (Tokyo, Japan).

### Generation of micro RNA-based knockdown cell lines under the control of a Tet-on system

Expression cassettes for artificial micro RNAs (miRNAs) targeting NLRP3, ASC, and MyD88 and for negative control miRNAs were generated using the BLOCK-iT Pol II miR RNAi Expression Vector Kit (Life Technologies, Carlsbad, CA, USA) and the following target sequences: for NLRP3, 5′-TCCTGAATCAGACTGAAGGCT-3′; for ASC, 5′-TAGGTCTCCAGGTAGAAGCTG-3′; and for MyD88, 5′-TCTCCAAGTACTCAAAGTCCA-3′. The DNA fragment containing the microRNA expression cassette was cloned into a Tet-on lentiviral vector pLVCT-tTRKRAB (Addgene, Cambridge, MA, USA) and transferred into HEK293FT packaging cells using the packaging vectors pMD2G and psPAX2. Recombinant lentiviruses collected from the culture supernatant were used to transduce THP-1 cells. We used vector-coded GFP to select THP-1 cells that expressed pLVCT-tTRKRAB-derived miRNA. The collected cells were cultured without Dox or treated with Dox for 5 d to induce miRNA expression.

### Generation of short hairpin (sh) RNA-based knockdown cell lines

The target sequence for NLRP3 (5′-GCTGGAATTGTTCTACTGTTT-3′) and a negative control sequence (5′-CCTAAGGTTAAGTCGCCCTCG-3′) were used to generate oligonucleotide pairs that were inserted into the pLKO.1 TRC cloning vector (Addgene) to produce lentiviral vectors that were used to transduce THP-1 cells. THP-1 cells stably expressing shRNA were selected by puromycin resistance for 2 weeks, and these cells were used for experiments.

### Cell culture and infection with Staphylococcus aureus (S. aureus)

The THP-1 cell line (ATCC TIB-202) and its derivatives expressing miRNA or shRNA were cultured in RPMI 1640 medium supplemented with 10% FCS. To induce IL-1β release, 1x10^5^ cells were primed with LPS (20 ng/ml) for 12 h and then with ATP (3 mM) for 1 h. Unprimed THP-1 and THP-derived cells were used for bacterial-infection and MSU-stimulation experiments. For bacterial-infection experiments, *S*. *aureus* (strain Smith, kindly provided by Dr, Nakanishi, Kanazawa University, Ishikawa, Japan) was grown in Luria broth until OD 1.9–2.2 at 600 nm, then collected and washed in RPMI 1640 with 10% FCS.

### Isolation of primary human monocytes

50 ml of fresh blood from a healthy donor was fractionated using 1.074 g/ml density barrier solution as described [26]. A monocyte-enriched fraction (purity > 80%) was prepared using Pan Monocyte Isolation Kit (Milteny Biotec K.K., Tokyo, Japan).

### Transfection of siRNAs

The NLRP3-targeted (HSS132811), MyD88-targeted (#428431 and #42832), ASC-targeted (HSS147064), and control siRNA (12395-115 and 12395-113) were purchased from Life Technologies. THP-1 and primary human monocyte were transfected with siRNA (20 nM) using the Neon Transfection System (Life Technologies).

### Western blot analysis

Immunoblotting was performed as described previously [27]. A hybridoma producing mAb against human NLRP3 was established using lymph node cells from mice immunized with recombinant NLRP3 (amino-acids 1–266). An anti-human ASC mouse mAb was prepared as described previously (27). Anti-p38 rabbit polyclonal Ab, anti-p54/p46 (JNK) rabbit mAb (56G8), anti-p44/p42 (ERK) rabbit mAb (137F5), anti-phospho-p-38 rabbit polyclonal Ab, anti-phospho-p54/p46 (JNK) rabbit mAb (81E11), and anti-phospho-p44/p42 (ERK) rabbit mAb (D13.14.4E) were purchased from Cell Signaling Technology (Beverly, MA). Anti-human IL-1β mouse mAb (8516) were purchased from R&D Systems (Minneapolis, MN). Anti-β-actin mouse mAb (AC-15) was purchased from Sigma-Aldrich.

### ELISA

The amount of human TNF-α, IL-1β, and IL-8 in culture supernatants was determined using the OptEIA ELISA kit (BD Pharmingen, Tokyo, Japan) according to the manufacturer’s protocol.

### EMSA

EMSA was performed as described previously [28].

### RT-PCR

Real-time PCR was performed with primers for TNF-α (forward 5′-ATGAGCACTGAAAGCATGATCC-3′, reverse 5′-GAGGGCTGATTAGAGAGAGGTC-3′), IL-1β (forward 5′-CTCGCCAGTGAAATGATGGCT-3′, reverse 5′-GTCGGAGATTCGTAGCTGGAT-3′), and MyD88 (forward 5′-CCCCAGCGACATCCAGTTT-3′, reverse 5′-GGCACCTCTTTTCGATGAGC-3′). Semiquantitative RT-PCR was performed as described previously [23] with primers for TNF-α (forward 5′-AAGGACACCATGAGCACTGA-3′, reverse 5′-CGTTTGGGAAGGTTGGATGTT-3′), IL-1β (forward 5′-AGCTGAGGAAGATGCTGGTT-3′, reverse 5′-CCAGGAAGACGGGCATGTTT-3′), and MyD88 (forward 5′-GACCCCCTGGGGCAT-3′, reverse 5′-TCAGGGCAGGGACAA-3′).

### Statistical analysis

All experiments were done in duplicate or triplicate and repeated at least three times. Error bars indicate standard deviations. Statistical significance of difference between two experimental groups was accessed by two-tailed Student’s t-test. *P*-values are indicated in the text and figures above the two groups. *P* <0.05 was considered statistically significant.

## Results

### NF-κB activation and cytokine induction by S. aureus infection were reduced in NLRP3- and ASC-knockdown cell lines

**Fig 1:**
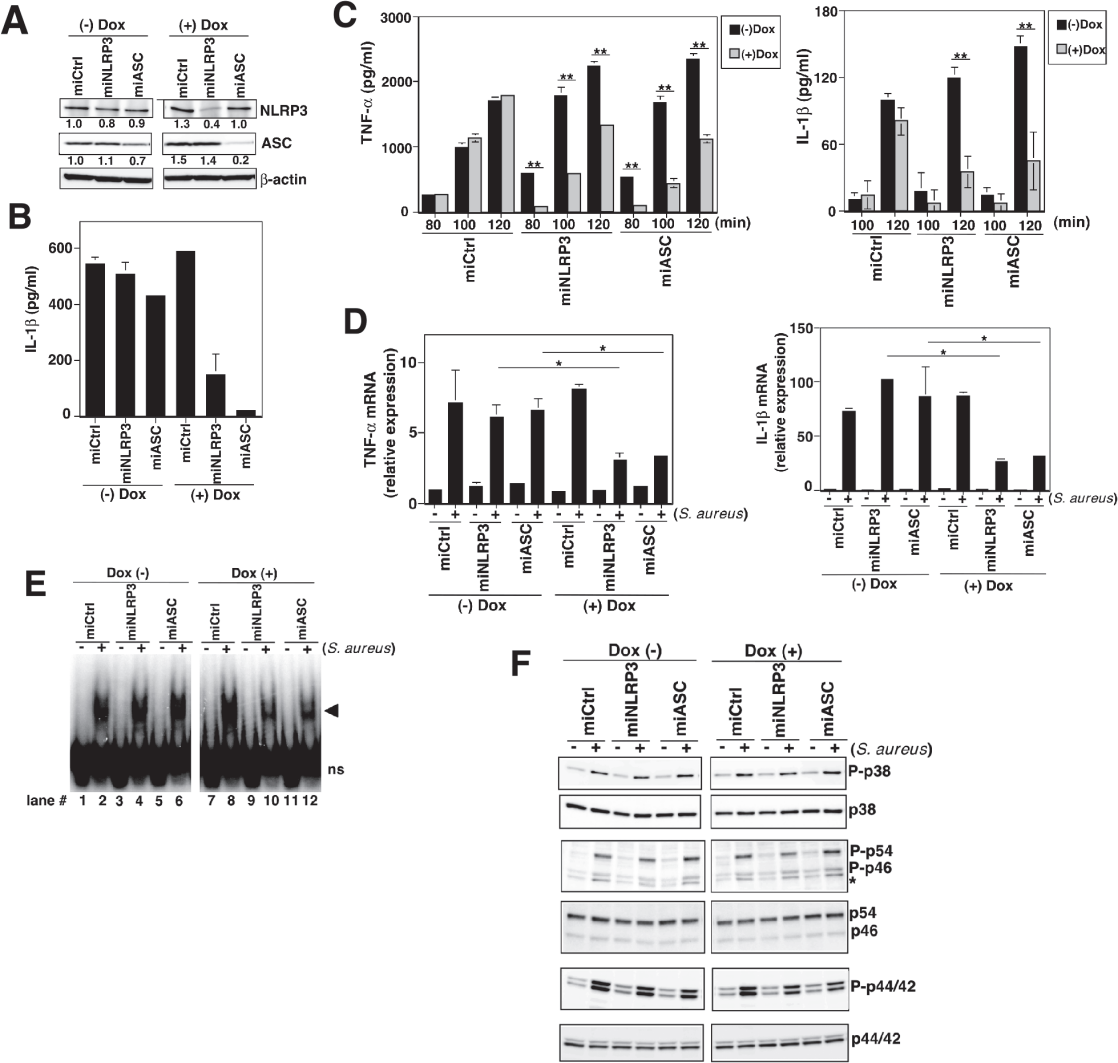
NF-κB activation and cytokine induction in *S*. *aureus*–infected THP-1 cells are reduced by NLRP3-knockdown. (A) THP-1 cells containing negative control miRNA (miCtrl), NLRP3 miRNA (miNLRP3), or ASC miRNA (miASC) were cultured with or without 2 ng/ml Dox for 5 d. Cells were collected and analyzed by Western blot with anti-NLRP3, anti-ASC, and anti-β-actin Abs. Numbers below lanes indicate band intensities relative to Dox-untreated control cells. (B) The indicated cell lines were stimulated with LPS (20 ng/ml) for 12 h and then pulsed with ATP (3 mM) for 1 h. IL-1β release was analyzed by ELISA. (C) ELISA analysis of TNF-α and IL-1β release in Dox-treated or –untreated cell lines infected with *S*. *aureus* at a multiplicity of infection (MOI) of 4 for the time periods indicated. Data are mean ± s.d. of triplicate samples. (D) Real-time PCR analysis of TNF-α and IL-1β mRNA in Dox-treated or -untreated cell lines infected with *S*. *aureus* at an MOI of 2 for 80 min. Values are relative to an average in uninfected, Dox-untreated miCtrl cells (set as 1). Values represent the averages of duplicate wells, and error bars represent the range. (E) Cells treated with or without Dox were infected with *S*. *aureus* at an MOI of 2 for 1 h, and NF-κB activation was examined by EMSA. The positions of nonspecific bands (ns) and shifted bands (arrowhead) are indicated. (F) Western blot analysis of p-p38, p-p54/p46 (JNK), and p-p44/p42 (ERK) in Dox-treated or -untreated cell lines infected with *S*. *aureus* at an MOI of 2 for 1 h. All results are representative of three independent experiments. *P<0.05, **P<0.01.

To determine NLRP3's role in activating NF-κB in human monocytes, we generated Dox-inducible, artificial miRNA-based NLRP3- or ASC-knockdown cell lines (miNLRP3 and miASC, respectively) by transducing human monocytic THP-1 cells with Tet-on-regulated lentiviral vectors expressing miRNA against NLRP3 or ASC. Before Dox treatment, the NLRP3 and ASC protein levels in miNLRP3 and miASC cells, respectively, were the same or slightly lower than those in cell lines transduced with negative-control miRNA (miCtrl) (Fig. 1A). After Dox treatment, the NLRP3 and ASC protein levels were markedly reduced in miNLRP3 cells and miASC cells, respectively, but were unaffected in miCtrl cells. In accordance with these results, the IL-1β secretion induced by LPS plus ATP was also reduced in the Dox-treated miNLRP3 and miASC cells (Fig. 1B), further confirming the effectiveness of the target-protein knockdown by this system.

We used the same cell lines to examine NLRP3's role in activating NF-κB and inducing cytokines in response to *S*. *aureus* infection [13]. In the Dox-treated NLRP3- and ASC-knockdown cells, not only IL-1β but also TNF-α and IL-8 secretion was dampened through 120 min after *S*. *aureus* infection (Fig. 1C and Fig. A in S1 File), although TNF-α and IL-8 levels in miNLRP3 and miASC cell culture supernatants were comparable in Dox-treated and -untreated cells at a later time point, 180 min after *S*. *aureus* infection (Fig. B in S1 File). The TNF-α and IL-1β mRNA induction in response to *S*. *aureus* infection was comparable among all cell lines before Dox treatment (Fig. 1D). In contrast, the induction of TNF-α and IL-1β mRNA was markedly reduced in Dox-treated miNLRP3 and miASC cells, but not in miCtrl cells. The effect of NLRP3 knockdown on cytokine induction in early stages of *S*. *aureus* infection was reproduced by experiments using shRNA-based NLRP3-knockdown cells (Fig. C in S1 File).

EMSA evaluation of the NF-κB activation after *S*. *aureus* infection showed comparable shifted bands corresponding to the NF-κB-DNA complex in the nuclear extracts from all cell lines that were not treated with Dox (Fig. 1E, left panel, lane 2, 4, and 6), indicating similar NF-κB activation in these cell lines. In contrast, Dox treatment (Fig. 1E, right panel, lane 10 and lane 12) diminished the NF-κB activation in cells expressing miNLRP3 or miASC. In miCtrl cells, NF-κB was activated at comparable levels before and after Dox treatment (lanes 2 and 8).

MAPK pathways also contribute to cytokine production in response to bacterial infection. In fact, MAPKs (p38, JNK, and ERK) were activated early in *S*. *aureus* infection in the context used in this study (Fig. 1F). However, NLRP3 and ASC knockdown did not affect p38, JNK, or ERK phosphorylation, suggesting that the MAPK pathway is independent of the NLRP3-ASC pathway.

Together, these results indicate that NLRP3 plays an important role in activating NF-κB and inducing cytokine genes in THP-1 cells in response to microbial infection.

### *TLRs redundantly activate N*F-*κB at an early stage of S*. *aureus infection*

**Fig 2:**
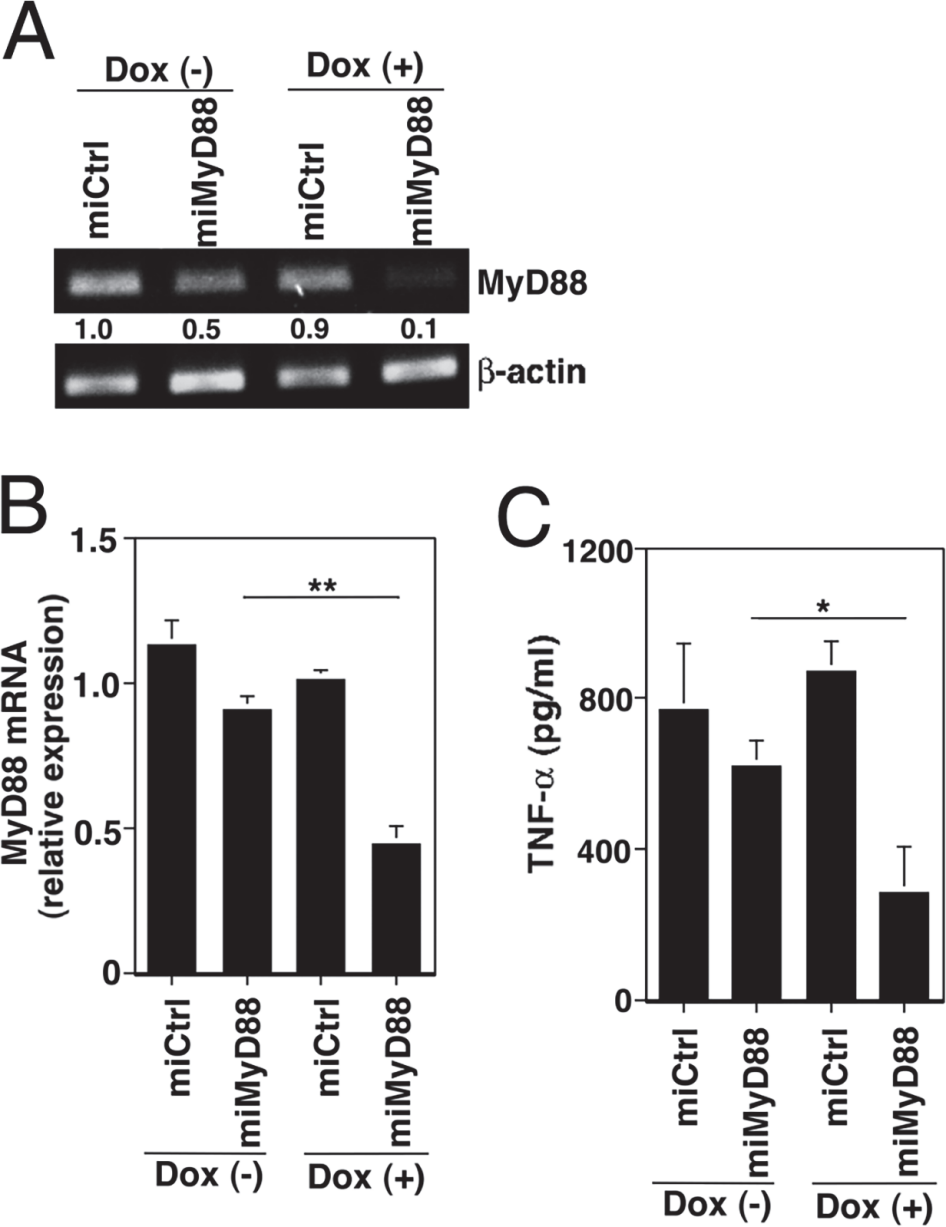
TLRs are involved in activating NF-κB in early *S*. *aureus* infection. (A and B) Control cells (miCtrl) and MyD88 miRNA-introduced cells (miMyD88) were cultured with or without 2 ng/ml Dox for 5 d. The total RNA was analyzed for MyD88 mRNA by RT-PCR (A) and real-time PCR (B); numbers below lanes indicate each band's intensity relative to a Dox-untreated control. (C) ELISA analysis of TNF-α released from each Dox-treated or -untreated cell line infected with *S*. *aureus* at an MOI of 4 for 120 min. Values represent the averages of duplicate wells, and error bars represent the range. All results are representative of three independent experiments. *P<0.05, **P<0.01.

When TLRs recognize bacterial ligands, they activate NF-κB to provoke the release of proinflammatory cytokines. To examine whether the TLR pathway is also involved in activating NF-κB and releasing cytokines in the early stages of infection, we generated MyD88-knockdown cell lines based on a Tet-on system. MyD88 mRNA was slightly reduced in the MyD88 miRNA-introduced (miMyD88) cells even before Dox treatment, and was reduced further by Dox treatment (Fig. 2A and Fig. 2B). Following *S*. *aureus* infection in the Dox-treated MyD88-knockdown cells, TNF-α release evaluated by ELISA (Fig. 2C) and NF-κB activation evaluated by EMSA (data not shown) were lower than in Dox-untreated cells. Thus, the NLPR3 and TLR signaling pathways seem to mediate NF-κB activation redundantly or cooperatively in the early stages of *S*. *aureus* infection.

### *NLRP3 mediates NF*-κ*B activation in sterile inflammation*

**Fig 3:**
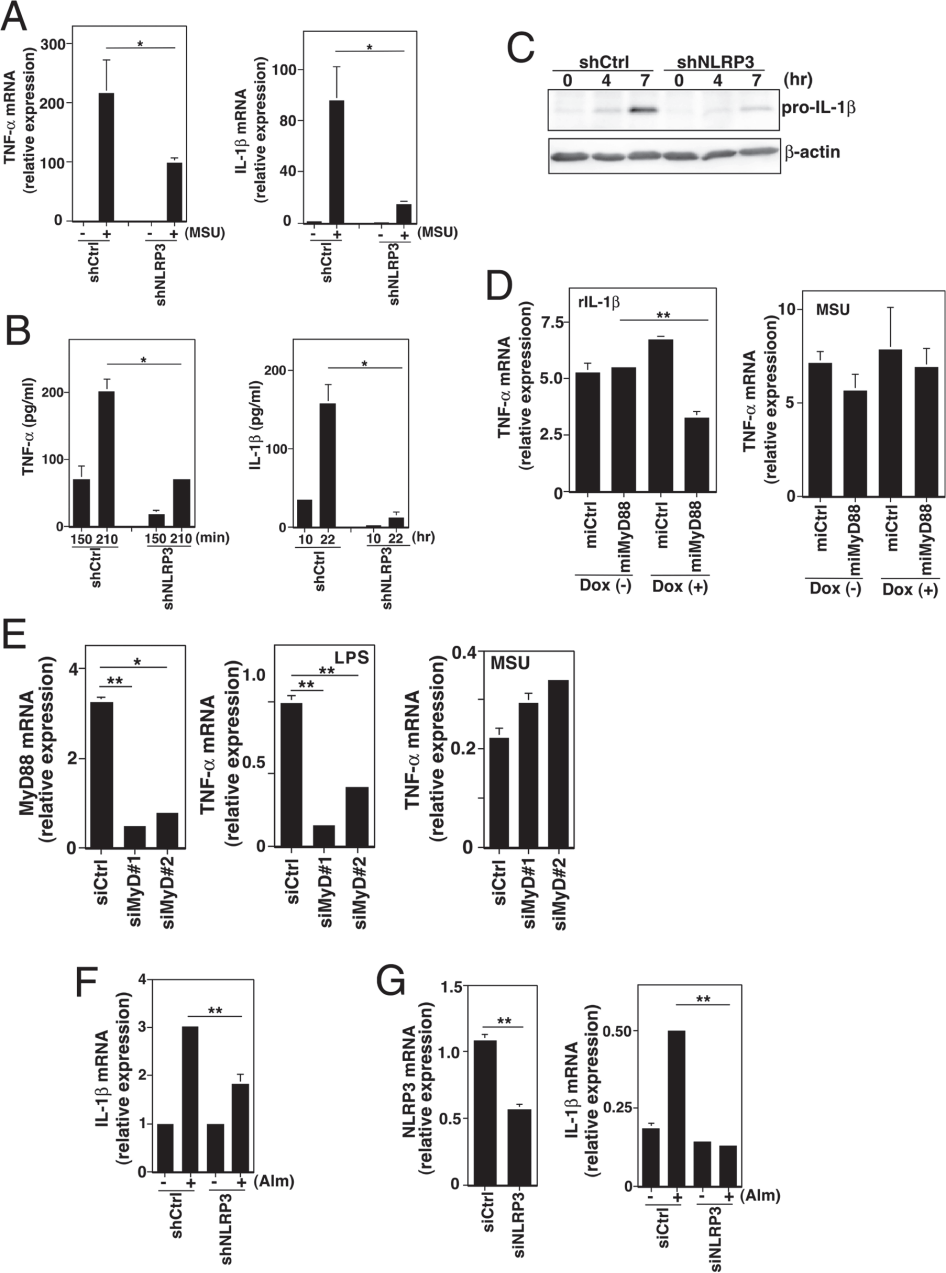
NLRP3 mediates NF-κB activation in sterile inflammation. (A and B) Real-time PCR analysis (A) and ELISA analysis (B) of TNF-α and IL-1β in shCtrl and shNLRP3 cell lines treated with 150 μg/ml MSU crystals for 90 min (A) or indicated period (B). (C) Immunoblot analysis of pro-IL-1β in cell lines treated with MSU crystals for the times indicated. (D) Real-time PCR analysis of TNF-α mRNA in cells treated with recombinant human IL-1β (1 ng/ml) or MSU crystals for 90 min. Values are normalized to an average of 1 in untreated cells. (E) Real-time PCR analysis of MyD88 (left panel) and TNF-α mRNA (middle and right panel) in negative-control siRNA-introduced (siCtrl) and MyD88 siRNA-introduced (siMyD#1, #2) cells left untreated (left panel) or treated with 10 ng/ml LPS (middle panel) or 150 μg/ml MSU crystals (right panel) for 60 min. (F) Real-time PCR analysis of IL-1β mRNA in shCtrl and shNLRP3 cell lines treated with 250 μg/ml aluminum adjuvants (Alm) for 140 min. (G) Real-time PCR analysis of NLRP3 (left panel) and IL-1β mRNA (right panel) in negative-control siRNA-introduced (siCtrl) and NLRP3 siRNA-introduced (siNLRP3) primary human monocytes treated with (right panel Alm +) or without (left panel, and right panel Alm −) 250 μg/ml of aluminum adjuvant for 140 min. All results are representative of at least three independent experiments. *P<0.05, **P<0.01.

To further investigate the pathophysiological significance of the NLRP3-mediated NF-κB activation, we examined the effect of NLRP3 knockdown on uric acid-induced inflammatory responses. MSU-induced TNF-α and IL-1β mRNA was reduced in the NLRP3-knockdown (shNLRP3) cells compared to control (shCtrl) cells (Fig. 3A). Consistent with this result, MSU-induced TNF-α and IL-1β secretion and intracellular pro-IL-1β accumulation were markedly reduced in shNLRP3 cells (Fig. 3B and Fig. 3C). The involvement of NLRP3 and ASC in MSU-induced TNF-α and IL-1β expression was confirmed using NLRP3 and ASC-targeting siRNAs, respectively (Fig. D in S1 File). Knocking down MyD88 by miRNA and siRNA reduced the IL-1β- and LPS-induced but not the MSU-induced TNF-α mRNA, indicating that the TLR pathway is not involved in the MSU-induced NF-κB activation (Fig. 3D and Fig. 3E). These results also rule out the possibility that contaminated LPS was responsible for the MSU-induced cytokine gene expression.

To further confirm the role of NLRP3 in sterile inflammation, we next examined the effect of NLRP3 knockdown on aluminum adjuvant-induced inflammatory responses. For this purpose, PMA-primed shCtrl and shNLRP3 cells were stimulated with aluminum adjuvant. In shNLRP3 cells, the extent of aluminum adjuvant-induced IL-1β mRNA induction was significantly lower than those in shCtrl cells (Fig. 3F). Similar results were obtained in the experiments using unprimed primary human monocytes (Fig. 3G). Together, these results indicate that NLRP3 plays an essential role in the NF-κB activation in sterile inflammation.

### No autocrine mechanism is involved in inducing cytokines in early stages of infection

**Fig 4:**
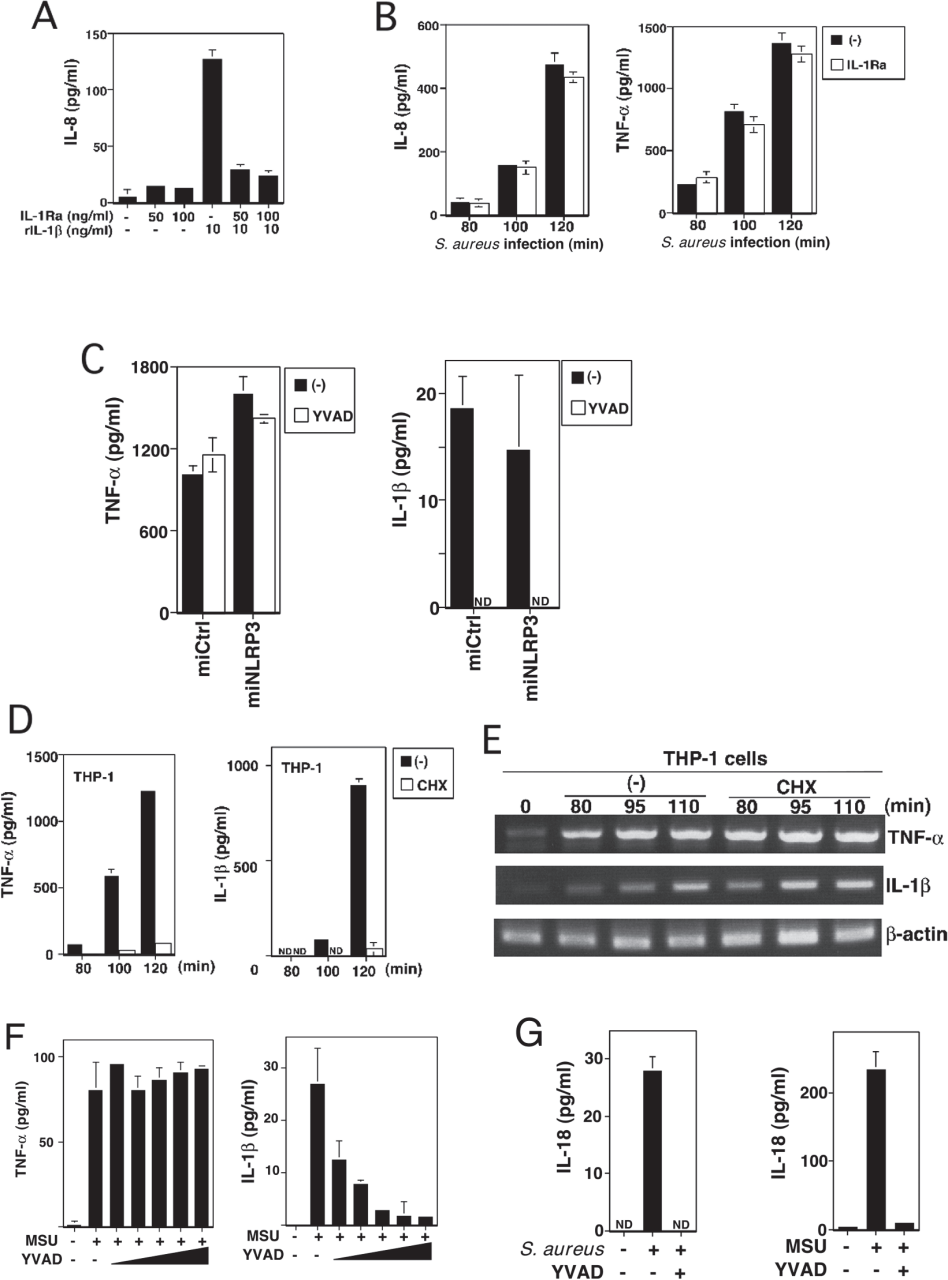
Cytokine induction in early infection does not involve an autocrine mechanism. (A) After incubation with the indicated amount of recombinant human IL-1Ra for 30 min, miCtrl cells were stimulated with recombinant human IL-1β for 60 min, and the IL-8 secretion was analyzed by ELISA. (B) After incubation with 100 ng/ml IL-1Ra for 30 min, miCtrl cells were infected with *S*. *aureus* at an MOI of 4 for the times indicated. IL-8 and TNF-α secretion from each cell line were analyzed by ELISA. (C) After incubation with 20 μM Ac-YVAD-CMK for 30 min, Dox-untreated miCtrl and miNLRP3 cells were infected with *S*. *aureus* for 100 min. TNF-α and IL-1β secretion were analyzed by ELISA. (D) THP-1 cells were incubated with 5 μg/ml CHX for 30 min and then infected with *S*. *aureus* at an MOI of 4 for the indicated times. TNF-α and IL-1β secretion were analyzed by ELISA. (E) THP-1 cells were treated with CHX and infected as in (D). The total RNA was analyzed for TNF-α and IL-1β mRNA by RT-PCR. (F) ELISA analysis of TNF-α and IL-1β released from THP-1 cells treated with MSU in the presence or absence of 0.63, 1.25, 2.5, 5, and 10 μM Ac-YVAD-CMK for 180 min. (G) ELISA analysis of IL-18 released from THP-1 cells treated as in (C) (left panel) or treated with MSU in the presence or absence of 10 μM of Ac-YVAD-CMK for 20 h (right panel). All results are representative of three independent experiments. ND, not detected.

The NLRP3-dependent NF-κB activation and cytokine mRNA induction noted above could have been mediated by quickly released IL-1β through an autocrine mechanism. However, this is unlikely because MyD88 knockdown inhibited the IL-1β-induced but not the MSU-induced TNF-α expression, as described above. To further examine whether the cytokines induced in early infection were mediated by quickly released IL-1β, THP-1 cells were pre-incubated with recombinant IL-1ra, which effectively suppresses the amount of IL-8 released in response to 10 ng/ml recombinant IL-1β, a dose 80–100 times higher than that induced by *S*. *aureus* infection (Fig. 4A). IL-1Ra-treated and -untreated cells secreted IL-8 and TNF-α at comparable levels in response to *S*. *aureus* infection (Fig. 4B). Consistent with these findings, the caspase-1 inhibitor Ac-YVAD-CMK, which completely blocks IL-1β and IL-18 secretion in response to *S*. *aureus* infection (Fig. 4C, right panel and Fig. 4G, left panel), did not affect TNF-α secretion (Fig. 4C, left panel). Furthermore, while CHX pre-incubation markedly reduced the TNF-α and IL-1β secretion in response to *S*. *aureus* infection (Fig. 4D), it did not inhibit TNF-α and IL-1β mRNA induction (Fig. 4E). These results indicate that the mRNA induction seen in early *S*. *aureus* infection is not mediated by an autocrine mechanism involving newly synthesized cytokines. In addition, the MSU-induced TNF-α secretion from THP-1 cells (Fig. 4F, left panel) and aluminum adjuvant-induced TNF-α and IL-1β mRNA expression from human peripheral blood monocytes (Fig. E in S1 File) were not affected by Ac-YVAD-CMK, which dampens MSU-induced IL-1β and IL-18 secretion (Fig. 4F, right panel and Fig. 4G, right panel). These results exclude the possible involvement of caspase-1-dependent secretion of cytokines including IL-1β and IL-18 in the induction of TNF-α.

### *NLRP3 mediates NF-*κ*B activation downstream of lysosomes*

**Fig 5:**
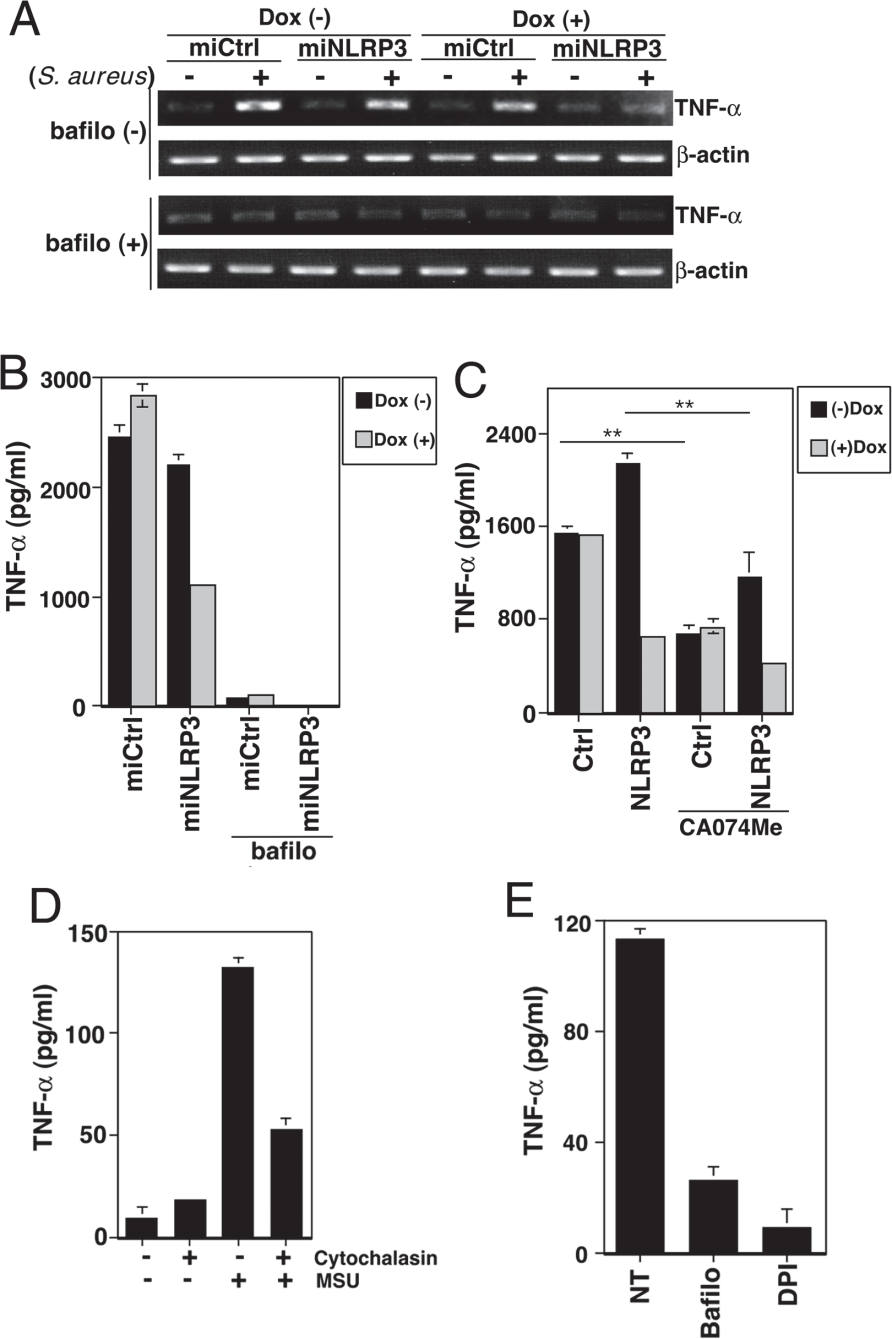
NLRP3 mediates NF-κB activation downstream of lysosomes. (A) After being cultured with or without Dox for 5 d, miCtrl and miNLRP3 cells were incubated with 500 nM bafilomycin A1 for 30 min and then infected with *S*. *aureus* at an MOI of 2 for 85 min. The total RNA was extracted and analyzed for TNF-α mRNA by RT-PCR. (B) ELISA analysis of TNF-α released from the indicated cell lines treated with or without bafilomycin A1 (bafilo) and then infected with *S*. *aureus* at an MOI of 4 for 120 min. (C) ELISA analysis of TNF-α released from the indicated Dox-treated or -untreated cell lines treated with or without CA-074Me (20 μM) for 30 min, and then infected with *S*. *aureus* at an MOI of 4 for 100 min. (D and E) ELISA analysis of TNF-α released from THP-1 cells treated with or without cytochalasin D (0.25 μM), bafilomycin A1 (0.5 μM), or DPI (2 μM), and then stimulated with MSU for 3 h. All results are representative of three independent experiments. **P<0.01.

To explore the signaling pathway by which NLRP3 activates NF-κB, we examined a proposed phagolysosome destabilization model for procaspase-1 activation [29, 30]. Pre-incubating control and knockdown cells with bafilomycin A1, which neutralizes the lysosomal pH and prevents lysosomal protease maturation, completely blocked the TNF-α mRNA induction and cytokine release (Fig. 5A and Fig. 5B) in the early stages of *S*. *aureus* infection. The cytokine release was markedly reduced by pre-incubating control and knockdown cells with the cathepsin B inhibitor CA-074Me (Fig. 5C, CA-074Me). Similarly, the MSU-induced TNF-α secretion was markedly reduced by pre-incubating cells with cytochalasin D, bafilomycin A1, or the NADPH oxidase inhibitor DPI (Fig. 5D and 5E). These results indicate that NLRP3 mediates NF-κB activation downstream of phagolysosome pathways and suggest that, in both sterile and microbially induced immune responses, NF-κB and inflammasomes may be activated concurrently.

## Discussion

In this study, we generated RNA-interference-based knockdown cells from the human monocytic THP-1 cell line, and demonstrated that NLRP3 is required for optimal NF-κB activation following *S*. *aureus* infection. Previous studies using macrophages from *NLRP3* ^−/−^ mice demonstrated that NLRP3 is dispensable for NF-κB activation following *S*. *aureus* infection [13]; this difference may be species-specific or may result from experimental conditions. In previous studies, mouse macrophages were primed with LPS, which strongly induces TLR4-mediated NF-κB activation, prior to bacterial infection; this would mask NLRP3-mediated NF-κB activation. In our experimental system, NLRP3-NF-κB activation could be detected only during early stages of infection (Fig. 1 and Fig. B in S1 File), probably because NF-κB is primarily activated through the TLR pathway at later stages of infection.

In sterile inflammatory diseases, signal 1 (for NF-κB activation leading to pro-IL-1β mRNA induction) has been unclear. It has been proposed that TLRs might mediate signal 1 by sensing DAMPs such as high mobility group box-1, hyaluronic acid, and biglycan, which are released from dead cells or extracellular matrix upon tissue injury [2, 31–35]. The data presented here show that NLRP3 is also able to mediate signal 1. Thus, NLRP3 mediates not only signal 2 but also signal 1 to produce IL-1β in sterile inflammation. In primary human monocytes, NLRP3 was also shown to mediate IL-1β mRNA induction. This could explain the spontaneous release of IL-1β by monocytes isolated from CAPS patients carrying gain-of-function NLRP3 mutations [19]. The smaller amounts and slower kinetics of cytokines induced by MSU (Fig. 3) compared with those induced by microbial infection (Fig. 1) may reflect the absence of TLR signaling in response to MSU.

Genetic reconstitution experiments with HEK293 cells have demonstrated that ASC can mediate NF-κB activation. ASC has also been reported to mediate NF-κB and MAPK activation following bacterial infection in THP-1 cells [25, 36]. However, one study concluded that NLRP3 is not required to activate MAPKs [36]. Whether ASC mediates the NLRP3-induced NF-κB activation under physiological conditions has not been clarified. Our knockdown experiments indicated that both NLRP3 and ASC are essential for the *S*. *aureus*-induced activation of NF-κB but not MAPKs, indicating that ASC mediates the NLRP3-induced NF-κB activation, but NLRP3 and ASC are not involved in MAPK activation under these conditions. The different results in terms of the involvement of ASC in MAPK activation may be due to different infectious agents between this and the previous study.

Our previous genetic reconstitution experiments using HEK293 cells demonstrated that caspase-8 is involved in ASC-mediated NF-κB activation [28, 37]. However, neither caspase-8 knockdown nor pretreatment with a caspase-8 inhibitor affected the *S*. *aureus*-induced NF-κB activation and cytokine gene expression in THP-1 cells (data not shown). Thus, at least one more mechanism exists for ASC-mediated NF-κB activation under these conditions. In this context, it should be note that NLRP3 and ASC have been reported to interact with MAVS and RIPK2, respectively [38–40], that could connect the NLRP3-ASC axis to NF-κB activation pathways. Further study is required to clarify how NLRP3 and ASC activated NF-κB in our experimental conditions.

Although NLRP3 has been linked to inflammasome in many microbially and sterile inflammatory responses, inflammasome-independent role for NLRP3 has also been reported [41–43]. In our study, pre-treatment with YVAD had no effect on cytokine induction in THP-1 and primary human monocytes (Fig. 4C, Fig. 4F, and Fig. E in S1 File). These suggest a novel caspase-1-independent pathway for NF-κB activation through NLRP3 in immune cells.

In summary, our results indicate that NLRP3 mediates the NF-κB activation in sterile inflammatory and microbially induced immune responses. So far, NLRP3 has been implicated in procaspase-1 activation through inflammasome formation. Our findings significantly broaden NLRP3's physiological importance in innate immunity: NLRP3 not only activates caspase-1 post-translationally, but also induces multiple cytokine genes, including *IL1B*.

## Supporting Information

S1 File Supporting figures. Fig. A, IL-8 secretion following *S*. *aureus* infection was dampened in Dox-treated NLRP3- and ASC-knockdown cells. Fig. B, NLRP3 and ASC was dispensable for TNF-α and IL-8 production at 180 min after *S*. *aureus* infection. Fig. C, Establishment of shRNA-based knockdown cells, and evaluation of TNF-α and IL-1β induction following *S*. *aureus* infection. Fig. D, The involvement of NLRP3 and ASC in MSU-induced TNF-α and IL-1β expression. Fig. E, Caspase-1 inhibitor Ac-YVAD-CMK did not inhibit aluminium adjuvant-induced TNF-α and IL-1β mRNA expression in primary human monocytes.
(PDF)
