# Supplementary material for: NLRP3 Mediates NF-κB Activation and Cytokine Induction in Microbially Induced and Sterile Inflammation
Source: PLoS One. 2015 Mar 11;10(3):e0119179. doi: 10.1371/journal.pone.0119179 (PMC4356585; doi:10.1371/journal.pone.0119179)
Supplement: S1 File — Fig. A, IL-8 secretion following S. aureus infection was dampened in Dox-treated NLRP3- and ASC-knockdown cells. Fig. B, NLRP3 and ASC was dispensable for TNF-α and IL-8 production at 180 min after S. aureus infection. Fig. C, Establishment of shRNA-based knockdown cells, and evaluation of TNF-α and IL-1β induction following S. aureus infection. Fig. D, The involvement of NLRP3 and ASC in MSU-induced TNF-α and IL-1β expression. Fig. E, Caspase-1 inhibitor Ac-YVAD-CMK did not inhibit aluminium adjuvant-induced TNF-α and IL-1β mRNA expression in primary human monocytes. (PDF) [file pone.0119179.s001.pdf]

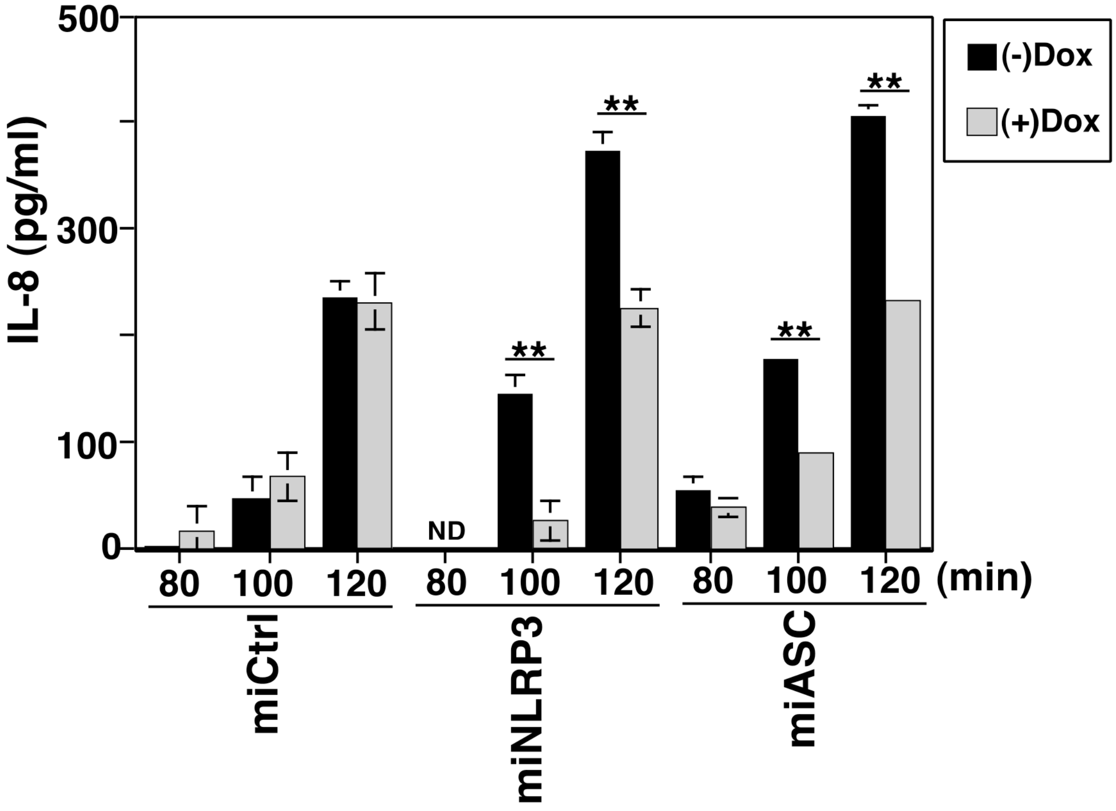

Figure A. IL-8 secretion following *S. aureus* infection was dampened in Dox-treated NLRP3- and ASC-knockdown cells.

The indicated cell lines were treated with or without Dox, and then infected with *S. aureus* as in Fig. 1. The IL-8 release was analyzed by ELISA. All results are representative of three independent experiments. Data are mean ± s.d. of triplicate samples. $**P<0.01$.

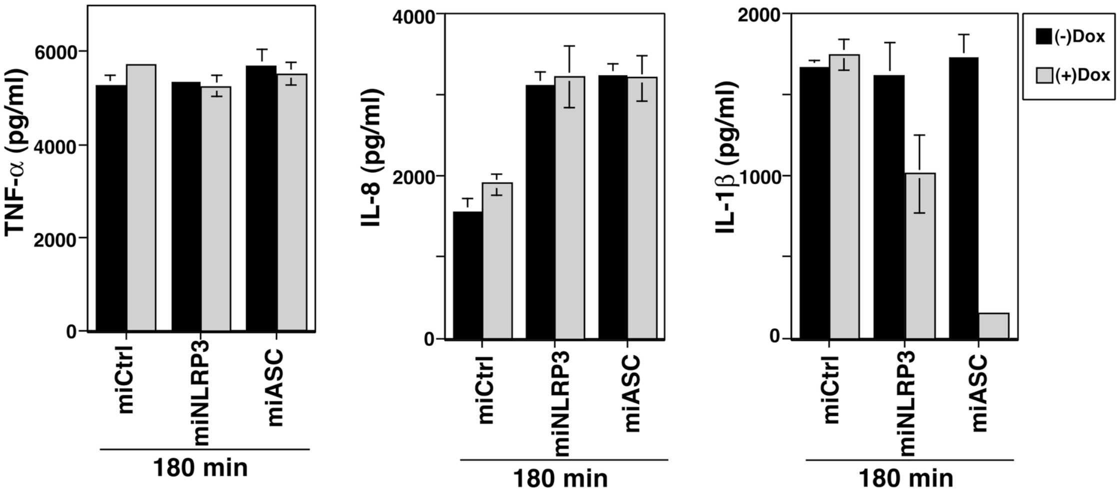

Figure B. NLRP3 and ASC were dispensable for TNF-α and IL-8 production at 180 min after *S. aureus* infection.

The indicated cell lines were treated with or without Dox, and then infected with *S. aureus* at an MOI of 4 for 180 min. TNF-α, IL-8, and IL-1β release were analyzed by ELISA. All results are representative of three independent experiments.

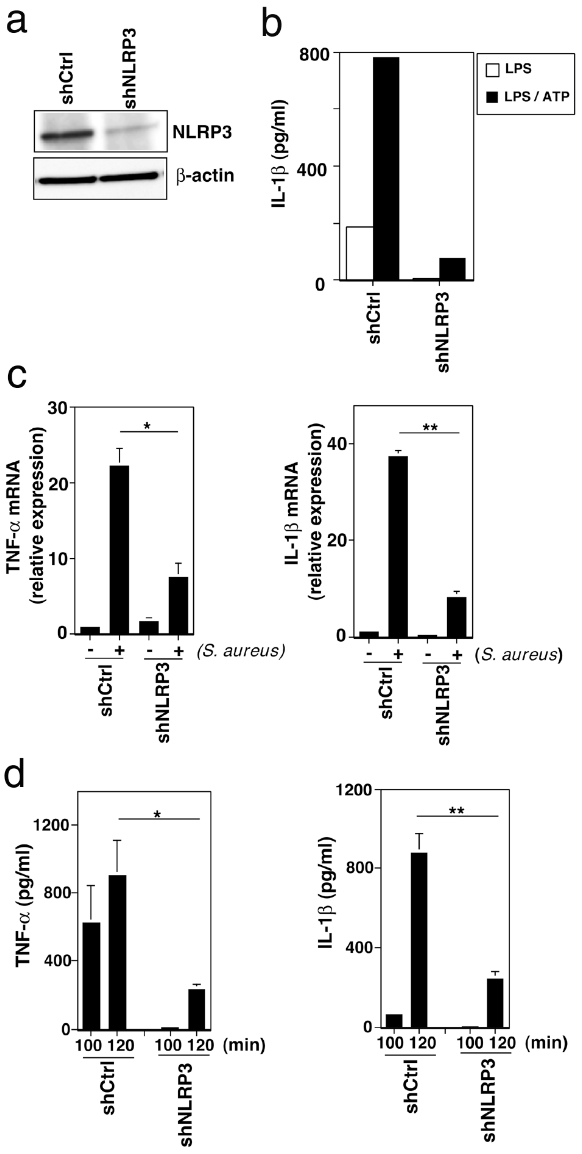

Figure C. Establishment of shRNA-based knockdown cells, and evaluation of TNF-α and IL-1β induction following *S. aureus* infection.

(a and b) Immunoblot analysis of NLRP3 in negative-control shRNA-introduced (shCtrl) and NLRP3 shRNA-introduced (shNLRP3) cells (a), and ELISA analysis of LPS+ATP-induced IL-1β secretion from these cell lines (b). (c) Real-time PCR analysis of TNF-α and IL-1β mRNA in the indicated cell lines after *S. aureus* infection at an MOI of 2 for 80 min. (d) ELISA analysis of TNF-α and IL-1β release from the indicated cell lines after *S. aureus* infection at an MOI of 4 for the indicated time periods. All results are representative of three independent experiments. *P<0.05, **P<0.01.

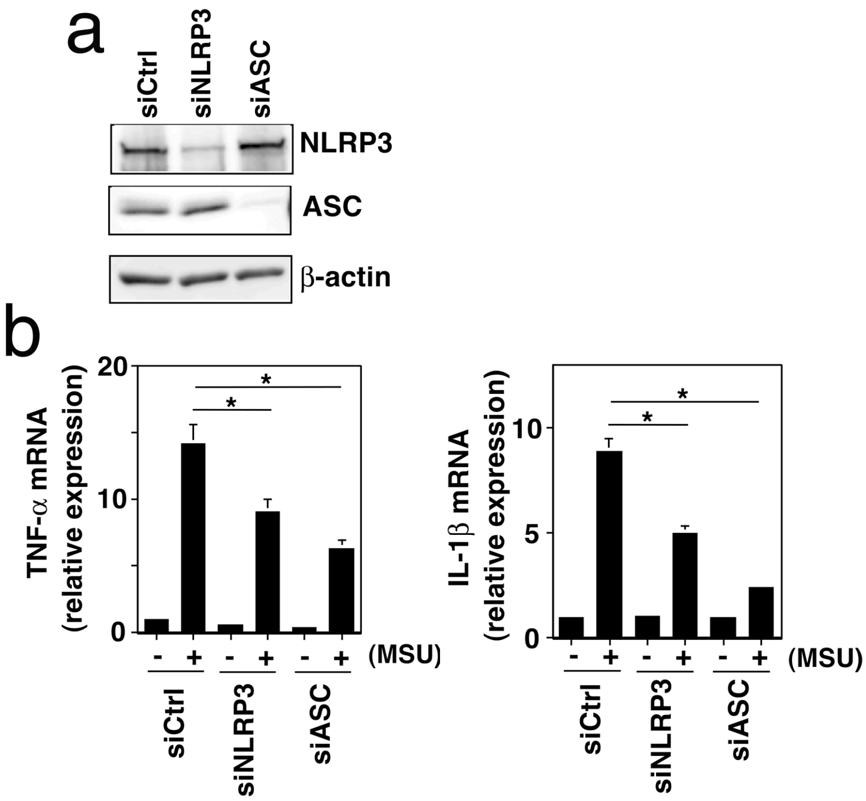

Figure D. The involvement of NLRP3 and ASC in MSU-induced TNF-α and IL-1β expression.

(a) Immunoblot analysis of NLRP3 and ASC in negative-control siRNA-introduced (siCtrl), NLRP3 siRNA-introduced (siNLRP3), and ASC siRNA-introduced cells (siASC). (b) Real-time PCR analysis of TNF-α and IL-1β mRNA in the indicated cells treated with 150 μg/ml MSU crystals for 100 min. All results are representative of three independent experiments. **P*<0.05.

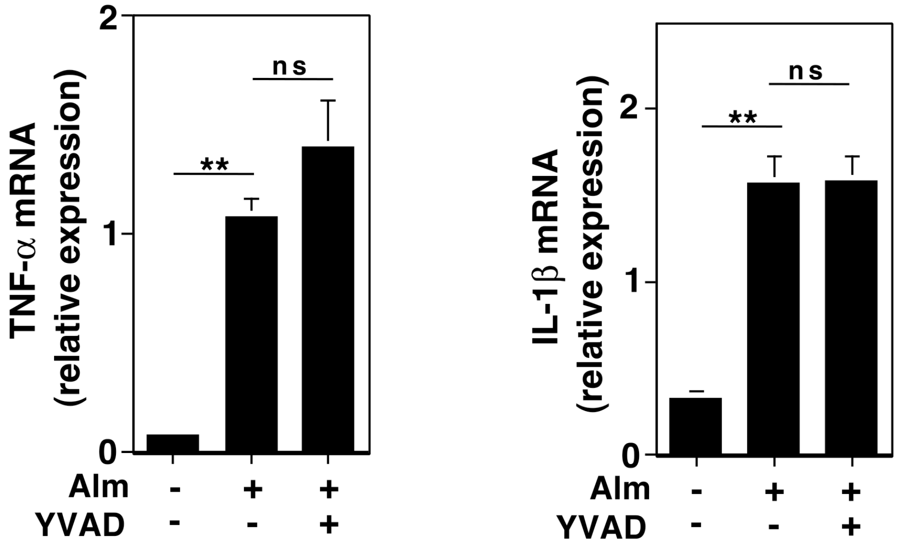

Figure E. Caspase-1 inhibitor Ac-YVAD-CMK did not inhibit aluminium adjuvant-induced TNF-α and IL-1β mRNA expression in primary human monocytes.

Real-time PCR analysis of TNF-α and IL-1β mRNA in primary human monocytes treated with or without 250 μg/ml of aluminium adjuvant in the presence or absence of 2 μM Ac-YVAD-CMK for 200 min. All results are representative of three independent experiments. *P<0.05. ns, non-significant.
